# MicroRNA-369 attenuates hypoxia-induced cardiomyocyte apoptosis and inflammation via targeting TRPV3

**DOI:** 10.1590/1414-431X202010550

**Published:** 2021-01-15

**Authors:** Jinghao Wang, Xu Chen, Wei Huang

**Affiliations:** 1 Harbin Medical University-Daqing, Department of Pharmacology, Daqing China Department of Pharmacology, Harbin Medical University-Daqing, Daqing, China; 2 the First Affiliated Hospital, Jinan University, Department of Pharmacy, Guangzhou China Department of Pharmacy, the First Affiliated Hospital, Jinan University, Guangzhou, China; 3 Daqing Oilfield General Hospital, Department of Pharmacy, Daqing China Department of Pharmacy, Daqing Oilfield General Hospital, Daqing, China

**Keywords:** miR-369, TRPV3, Hypoxia, Apoptosis, Inflammation

## Abstract

Hypoxia-induced apoptosis and inflammation play an important role in cardiovascular diseases including myocardial infarction (MI). miR-369 has been suggested to be a key regulator of cardiac fibrosis. However, the role of miR-369 in regulating hypoxia-induced heart injury remains unknown. Our data indicated that miR-369 expression was significantly down-regulated and TRPV3 was significantly up-regulated in myocardial tissue after MI in rats and in hypoxic-treated neonatal rat cardiomyocytes (NRCMs). In addition, we observed that hypoxia significantly promoted apoptosis and the inflammatory response, accompanied by increased caspase-3 activity and the secretion of the cytokines interleukin (IL)-6, IL-1β, and tumor necrosis factor (TNF)-α. miR-369 overexpression significantly suppressed cell apoptosis and inflammatory factor production triggered by hypoxia, whereas miR-369 inhibition had an opposite effect. Importantly, we identified TRPV3 as a direct target of miR-369-3p. TRPV3 inhibition with small interfering RNA (siRNA) significantly inhibited hypoxia-induced inflammation and apoptosis, which can reverse the injury effects of miR-369 inhibitors. Our findings indicated that miR-369 reduced hypoxia-induced apoptosis and inflammation by targeting TRPV3.

## Introduction

Acute myocardial infarction (MI) is the major cause of death in patients with cardiovascular disease (1). A hypoxic/ischemic myocardium undergoes a series of physical and chemical changes, as well as inflammation, apoptosis, necrosis, and myocardial remodeling (2). Indeed, growing evidence highlights the importance of inflammatory response and myocardial apoptosis in the pathogenesis of acute MI, as they are involved in mediating impaired myocardial function and heart failure (2,3). Immediately after MI, an inflammatory response is triggered. Although inflammatory cell infiltration can clear necrotic cardiomyocytes and extracellular matrix fragments to promote the healing process (2), continued inflammatory and immune infiltration is directly related to myocardial apoptosis and impairs cardiac function (4,5). MI-induced myocardial apoptosis persists for months after the acute phase following MI (6). The loss of cardiomyocytes in the border and remote areas of the infarct zone can lead to sustained damage to the structure and function of the heart, accounting for the progression to heart failure (7). A better understanding of the regulators involved in myocardial apoptosis and inflammation is important for the exploitation of novel therapeutic targets for MI.

MicroRNAs (miRNAs) are a class of highly conserved small (about 22 nucleotides) non-coding RNAs that play a key role in regulating gene expression by directly binding the 3′-UTR of their target gene mRNA, leading to translational inhibition or degradation (8). In recent years, more and more evidence shows that miRNAs play an important role in the occurrence and development of ischemic heart disease (9-11). A large number of studies indicate that targeting specific miRNAs, such as miR-145, miR-155, and miR-26b, can effectively attenuate ischemia/hypoxia-induced cardiomyocytes apoptosis and inflammation (12-14). In recent years, a study showed that miR-369 attenuated the proliferation of fibroblasts in cardiac fibrosis (15). However, the role of miR-369 in hypoxia-induced myocardial apoptosis and inflammation remains unclear. TRPV3 is a member of the transient receptor potential (TRP) channel family of non-selective cation channels, and they have been reported to be involved in the regulation of various diseases such as anxiety, asthma, obesity, and metabolic diseases (16). TRPV3 activation exacerbates cardiac fibrosis by promoting cardiac fibroblast proliferation and exacerbating pathological myocardial hypertrophy (17–19). However, the role and mechanism of TRPV3 in cardiomyocytes apoptosis and inflammation have not been studied.

In the current study, we used a hypoxia-induced cardiomyocytes injury model to investigate the effects of miR-369 on hypoxia-induced cardiomyocytes apoptosis and inflammation and determine the underlying mechanisms.

## Material and Methods

### Animal experiments

This study was conducted in accordance with the guidelines of the Animal Protection and Use Committee of Harbin Medical University. The protocol was approved by the Animal Experiment Ethics Committee of Harbin Medical University. In this study, male Wistar rats (220±20 g) were used. As we have previously described (20), the MI model was established by ligating the left anterior descending (LAD) coronary artery. Rats were anesthetized with ketamine-xylazine (100 mg/kg, 5 mg/kg, intraperitoneally). Sham-operated animals underwent the same procedure, but the coronary artery was not tied. Electrocardiograms were recorded before and after ligation to confirm ischemia.

### Measurement of infarct size

Three days after MI, the heart was stained with TTC (triphenyltetrazolium chloride, Sigma-Aldrich, USA), and the size of the infarct was measured. After the remaining blood was washed away, the heart was cut into 2-mm-thick sections below the ligature and stained with 1% TTC at 37°C for 15 min. Infarct area is stainless while the non-infarct area is stained red. The left ventricle was separated, and the ischemic area of the ventricle was dissected. The infarct area was measured by the weight ratio of infarct area and left ventricle.

### Caspase-3 and LDH activity assay

As described in previous studies, caspase-3 (Beyotime Institute of Biotechnology, China) and lactate dehydrogenase (LDH) activity kits (Nanjing Jiancheng Institute of Biotechnology, China) were used to determine caspase-3 and LDH activities (20).

### Cell culture and transfection

The culture process of neonatal rat cardiomyocytes (NRCMs) was as described previously (21). Briefly, the hearts of newborn Wistar rats were quickly removed, minced in serum-free Dulbecco's modified Eagle's medium (DMEM, HyClone, USA), and then digested in a 0.25% trypsin solution. The digested cells were placed in DMEM containing 10% fetal bovine serum (FBS, Hyclone, USA), centrifuged (425 *g*, 10 min, 37°C), placed in a culture flask (uncoated), and then 0.1 mM bromodeoxyuridine was added to the culture medium. Cardiac cells were cultured in 5% CO_2_ at 37°C.

Guangzhou Ribo Biological Co., Ltd. (China) synthesized miR-369 mimic (50 nM), miR-369 inhibitor (100 nM), and negative control miRNA (miR-NC, 50 nM). The sequence of the miR-369-3p is 5′-AAUAAUACAUGGUUGAUCUUU-3′ and NC is 5′-UUUGUACUACACAAAAGUACUG-3′. TRPV3 siRNA (100 nM) was purchased from Thermo Fisher Scientific (USA). Myocardial cells (1×10^5^/well) were starved in serum-free medium for 24 h before transfection with X-treme GENE siRNA transfection reagent (Roche, Germany) according to the manufacturer's instructions. Forty-eight hours after transfection, NRCMs were treated with 1% O_2_, 5% CO_2_, and 94% N_2_ for 12 h in a module incubator.

### Cell viability assay

Cells (2×10^4^ cells/well) were seeded in 96-well culture plates. Cell viability was measured by 3-(4,5-dimethylthiazol-2-yl)-2,5-diphenyltetrazolium bromide (MTT) assay according to the manufacturer's instructions. The absorbance was measured at 490 nm. Cell viability is reported as relative viable cells (%) that control cardiomyocytes.

### ELISA analysis

Cellular supernatant levels of IL-6, IL-1β, and TNF-α were measured by ELISA according to the manufacturer's instructions (R&D, USA). All measurements were performed four times.

### Terminal deoxynucleotidyl transferase-mediated dUTP nick-end labeling (TUNEL) assay

According to the manufacturer's instructions, apoptosis of NRCMs was detected by the TUNEL fluorescent FITC kit (Roche, USA). After TUNEL staining, the nuclear solution was stained with 4,6-dimidyl-2-phenylindole (1:100, Beyotime Biotechnology, China). Fluorescence staining was observed with a confocal laser scanning microscope (FV1000, Olympus, Japan). The apoptotic rate was calculated as TUNEL-positive cells per field.

### Quantitative real-time PCR

Total RNA was extracted from different processed heart tissue or myocardial cells using TRIZOL reagent (Invitrogen, USA) according to the manufacturer's protocol. Then, total RNA was reverse transcribed using TaqMan miRNA RT kit (Applied Biosystems; Thermo Fisher Scientific, Inc.) for 1 h at 37°C. U6 was used as an internal control to measure mRNA level with SYBR Green on the RocheLightCycler^®^ 480 Real-Time PCR System (Roche, USA). The sequences of the primers were miR-369-3p forward: 5′-TGACCCAAGGGACTCCCACAA-3′, reverse: 5′-TAGCAATATTGCACAGAAGGC-3′; U6 forward: 5′-GCTTCGGCACATATACTAAAAT-3′, reverse: 5′-CGCTTCACGAATTTGCGTGTCAT-3′. The target amount (2^-ΔΔCT^) was obtained by normalizing to the endogenous reference and relative to the calibrator (average of the control sample).

### Luciferase assay

To generate a reporter vector with a miRNA binding site, three untranslated regions (3′-UTR) of TRPV3 were synthesized by Sangon (China). The construct was inserted into multiple cloning sites (SacI and HindIII sites) downstream of the luciferase gene in the pMIR-REPORT luciferase miRNA expression reporter vector (Ambion, USA). For the luciferase assay, using Lipofectamine 2000 (Invitrogen), 0.1 μg of a luciferase reporter containing 3′-UTR was co-mingled with miR-369-3p mimics or miR-369-3p inhibitors or NC transfection into HEK-293 cells (Shanghai Institute of Cell Biology, China). As an internal control, 10 ng of Renilla luciferase reporter was also included. Forty-eight hours after transfection, cells were collected and double luciferase activity was measured by a luminometer according to the manufacturer's instructions (Promega Corporation, USA).

### Western blot analysis

Total protein samples were extracted from cardiac tissue or cardiomyocytes for western blot analysis (20). Protein samples (50 µg) were separated by SDS-PAGE (10% polyacrylamide gel) and transferred to a nitrocellulose membrane. The membrane was blocked in 5% non-fat milk in PBS for 2 h, and then overnight at 4°C with the following primary antibodies: TRPV3 (1:100, Abcam, USA) and β-actin (1:1000, Santa Cruz, USA). After washing, the membrane was stirred with the secondary antibody for 1 h. Images were captured on an Odyssey CLx infrared imaging system (LI-COR Biosciences, USA). Odyssey CLx v2.1 software was used to quantify western blot bands. Data were normalized to β-actin as an internal control.

### Data analysis

Group data are reported as means±SE. Statistical analysis was performed using one-way ANOVA followed by Tukey's test. Student's *t-*test was used for comparisons between two groups. When P<0.05, the difference was considered statistically significant.

## Results

### MiR-369 was down-regulated and TRPV3 up-regulated during ischemia/hypoxia treatment

**Figure 1:**
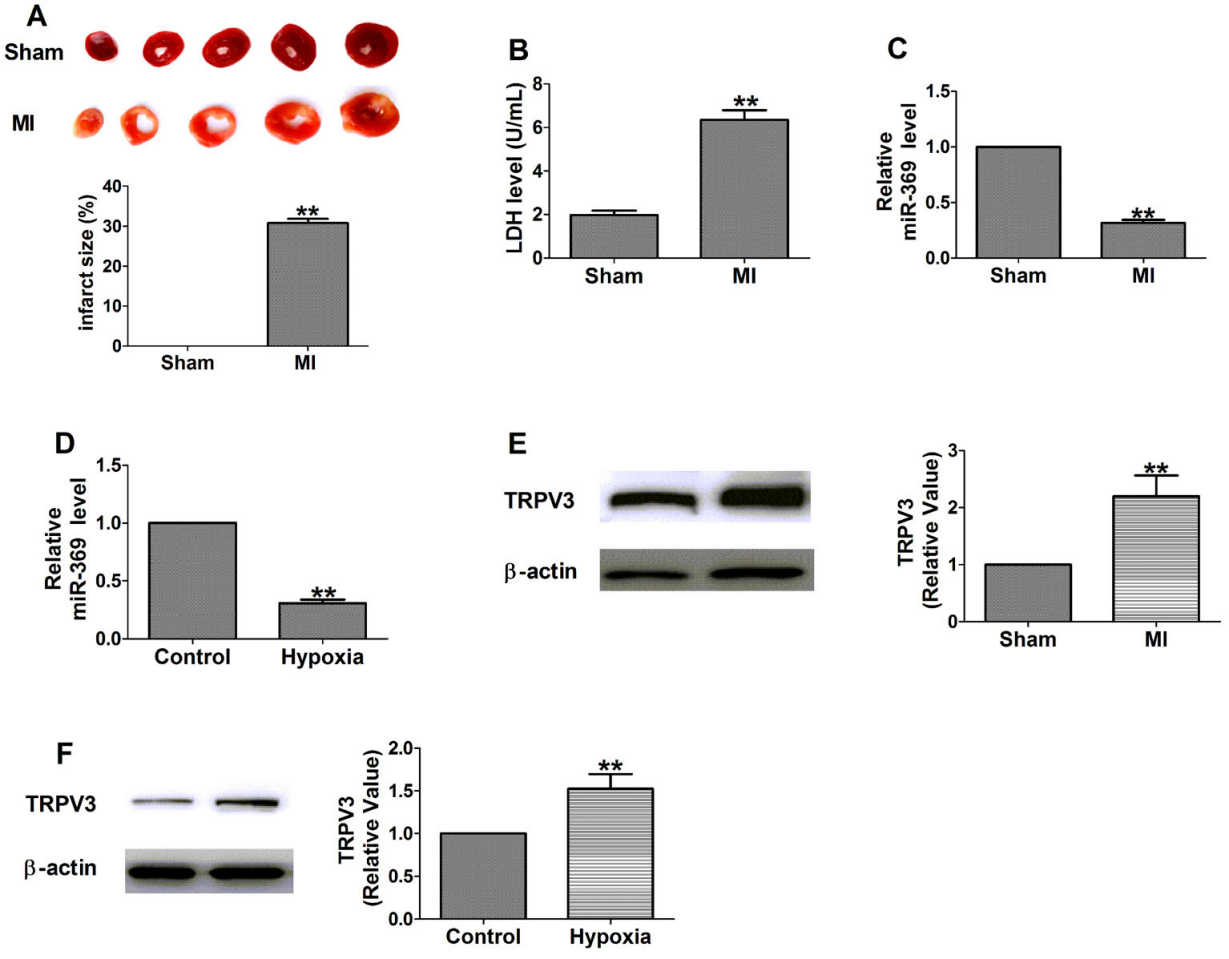
**A**, Representative images showing infarct areas in cross-section slices and statistical analysis of infarct area/left ventricles ratio (n=6). **B**, Serum lactate dehydrogenase (LDH) activity (n=4). **C** and **D**, Relative mRNA level of miR-369 (n=6). **E** and **F**, The expressions of TRPV3 protein (n=3). Data are reported as means±SE. **P<0.01 *vs* sham or control group (Student's *t*-test). MI: myocardial infarction.

We established a rat model of MI by obstructing the left anterior descending coronary arteries. The increase in the infarct size of the MI group indicated that the model was successfully established (Figure 1A). We found a significant increase in serum LDH activity at 3 days post-MI compared to the sham group (Figure 1B).

miR-369 expression was significantly down-regulated in ischemia/hypoxia-induced cardiomyocytes compared to the sham/control group (Figure 1C and D). Subsequently, we found that TRPV3 was significantly overexpressed in myocardial cells induced by ischemia/hypoxia (Figure 1E and F). The data indicated that miR-369 was down-regulated and TRPV3 was up-regulated in response to ischemia/hypoxia treatment.

### MiR-369 attenuated hypoxia-induced apoptosis in cardiomyocytes

**Figure 2:**
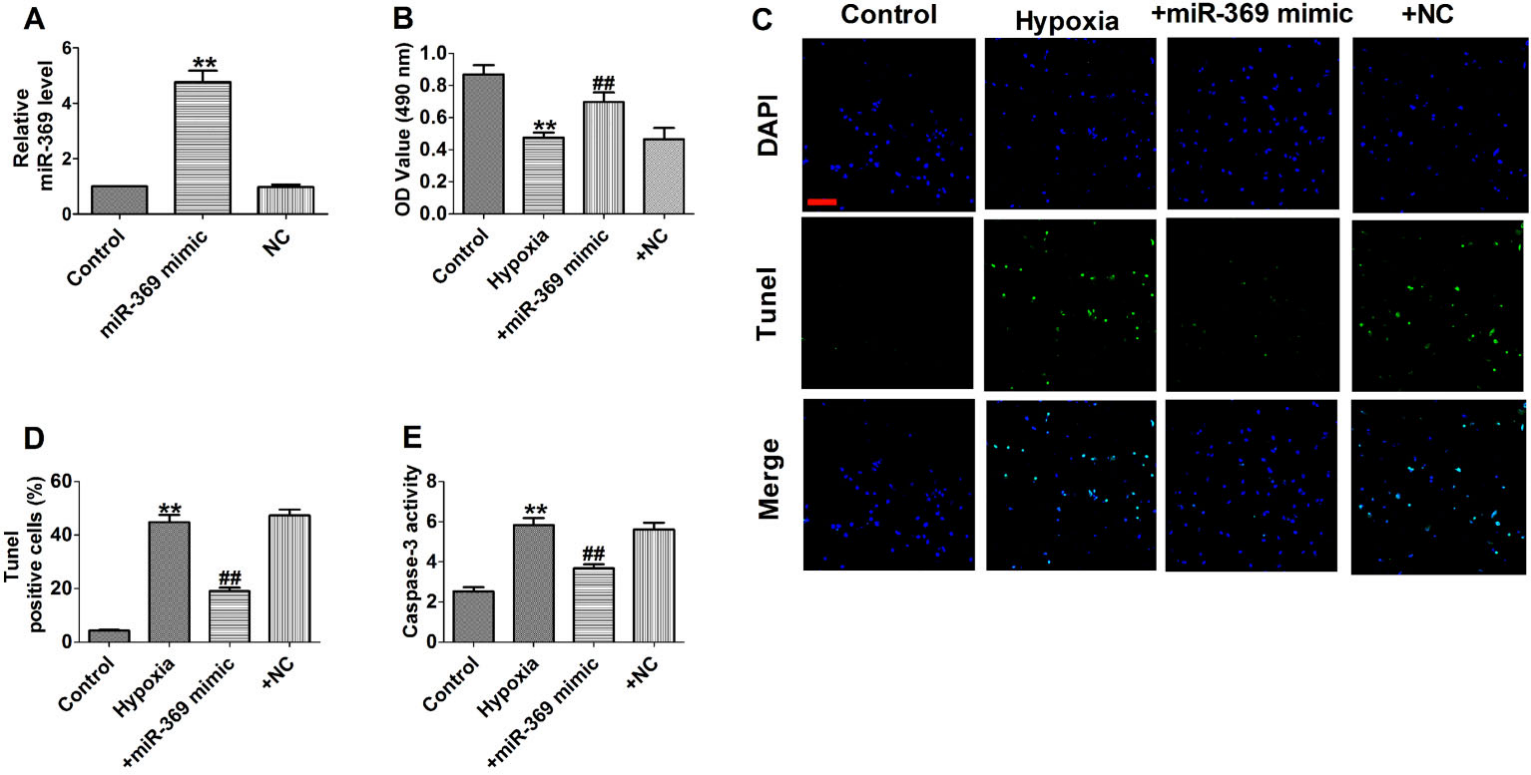
**A**, The relative mRNA level of miR-369 (n=6). **B**, MTT assay (n=6). **C**, Representative images of TUNEL staining of cardiomyocytes showing the apoptotic cells (100×; scale bar, 100 μm). **D**, Statistical results of TUNEL-positive cells per field (n=4). **E**, Caspase-3 activity (n=6). Data are reported as means±SE. **P<0.01 *vs* control group; ^##^P<0.01 *vs* hypoxia group (ANOVA). NC: negative control.

miR-369 mimic transfection significantly up-regulated miR-369 expression in cardiomyocytes (Figure 2A). MTT analysis showed that miR-369 overexpression significantly increased cell viability under hypoxic treatment of cardiomyocytes (Figure 2B). Next, miR-369 mimic significantly reduced hypoxia-induced apoptotic cells (Figure 2C and D). In addition, miR-369 overexpression also down-regulated caspase-3 activity in hypoxia-induced cardiomyocytes (Figure 2E). Overall, these results indicated that up-regulation of miR-369 attenuated hypoxia-induced cell apoptosis.

### MiR-369 attenuated hypoxia-induced inflammation in cardiomyocytes

**Figure 3:**
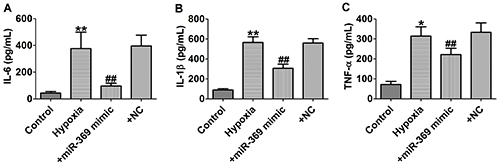
A-**C**, The concentrations of interleukin (IL)-6, IL-1β, and tumor necrosis factor (TNF)-α were measured by ELISA assay. Data are reported as means±SE for n=4. *P<0.05, **P<0.01 *vs* control group; ^##^P<0.01 *vs* hypoxia group (ANOVA). NC: negative control.

The concentrations of IL-6, IL-1β, and TNF-α inflammatory cytokines were increased significantly after hypoxia stimulation. However, this situation was eliminated by adding miR-369 mimic (Figure 3A-C). These findings indicated that overexpression of miR-369 inhibited hypoxia-induced inflammatory responses in cardiomyocytes.

### *TRPV3* is a direct target of miR-369

**Figure 4:**
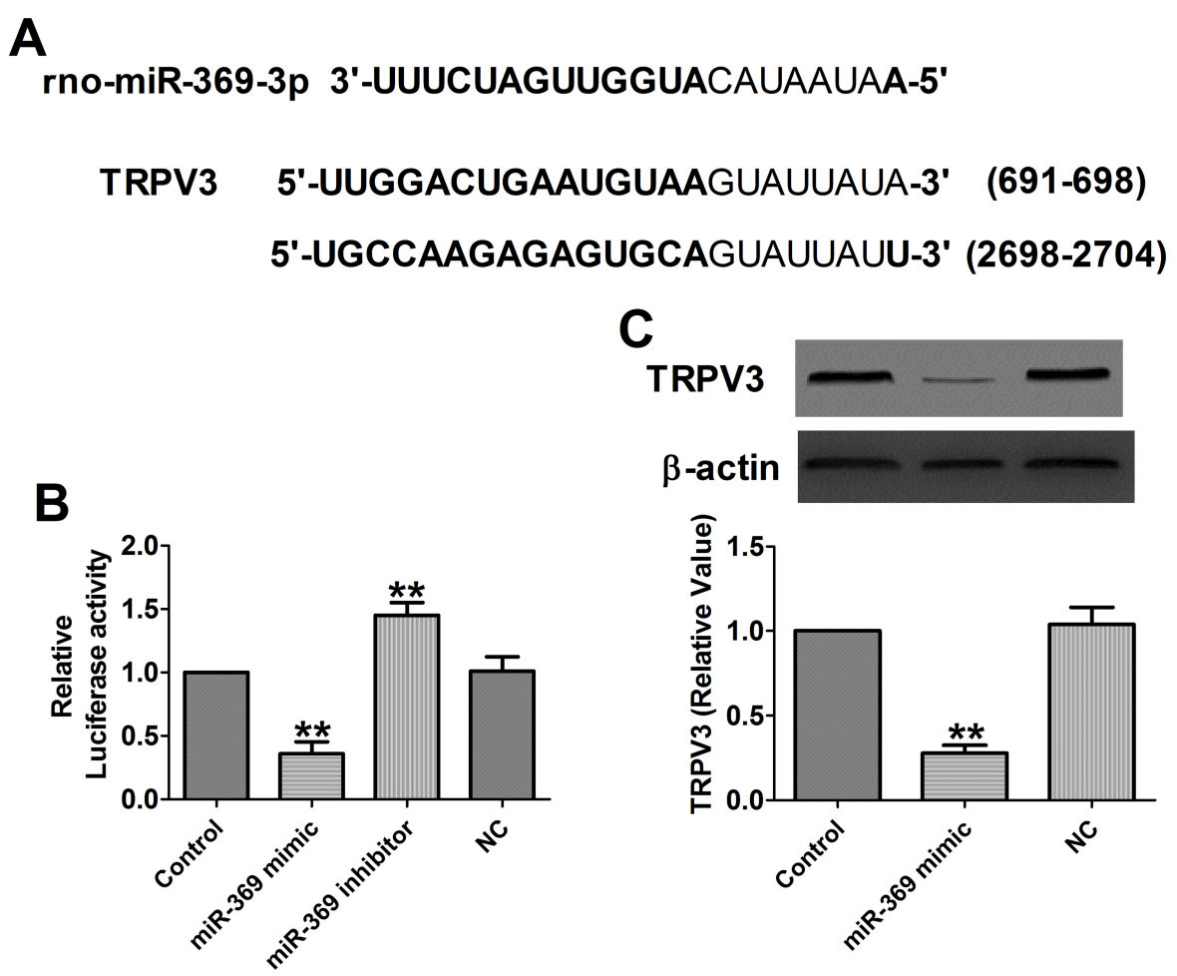
**A**, Sequence alignment between miR-369 and the binding sites in the 3’UTR of the TRPV3 gene. **B**, Interaction between miR-369 and its binding sites in the 3′UTR of TRPV3 was examined by luciferase assay in HEK293 cells (n=4). **C**, Expressions of TRPV3 protein (n=3). Data are reported as means±SE. **P<0.01 *vs* control group (*t*-test). NC: negative control.

Interestingly, we found that *TRPV3* was predicted to be one of the target genes of miR-369, and which is an important regulatory gene for cardiovascular disease (17–19) (Figure 4A). To validate this prediction, we performed a dual luciferase reporter assay. The results showed that miR-369 significantly inhibited the luciferase activity of the plasmid containing the 3′-UTR of TRPV3, while the inhibition of miR-369 significantly increased the luciferase activity (Figure 4B). To further prove whether *TRPV3* is a downstream regulatory target of miR-369, we used western blot to monitor the expression level of *TRPV3* in myocardial cells. The results showed that TRPV3 protein levels were significantly reduced after miR-369 overexpression (Figure 4C). Taken together, these results indicated that *TRPV3* is a direct target gene of miR-369-3p.

### TRPV3 contributed to the effect of miR-369 on hypoxia-induced cell apoptosis and inflammatory response

**Figure 5:**
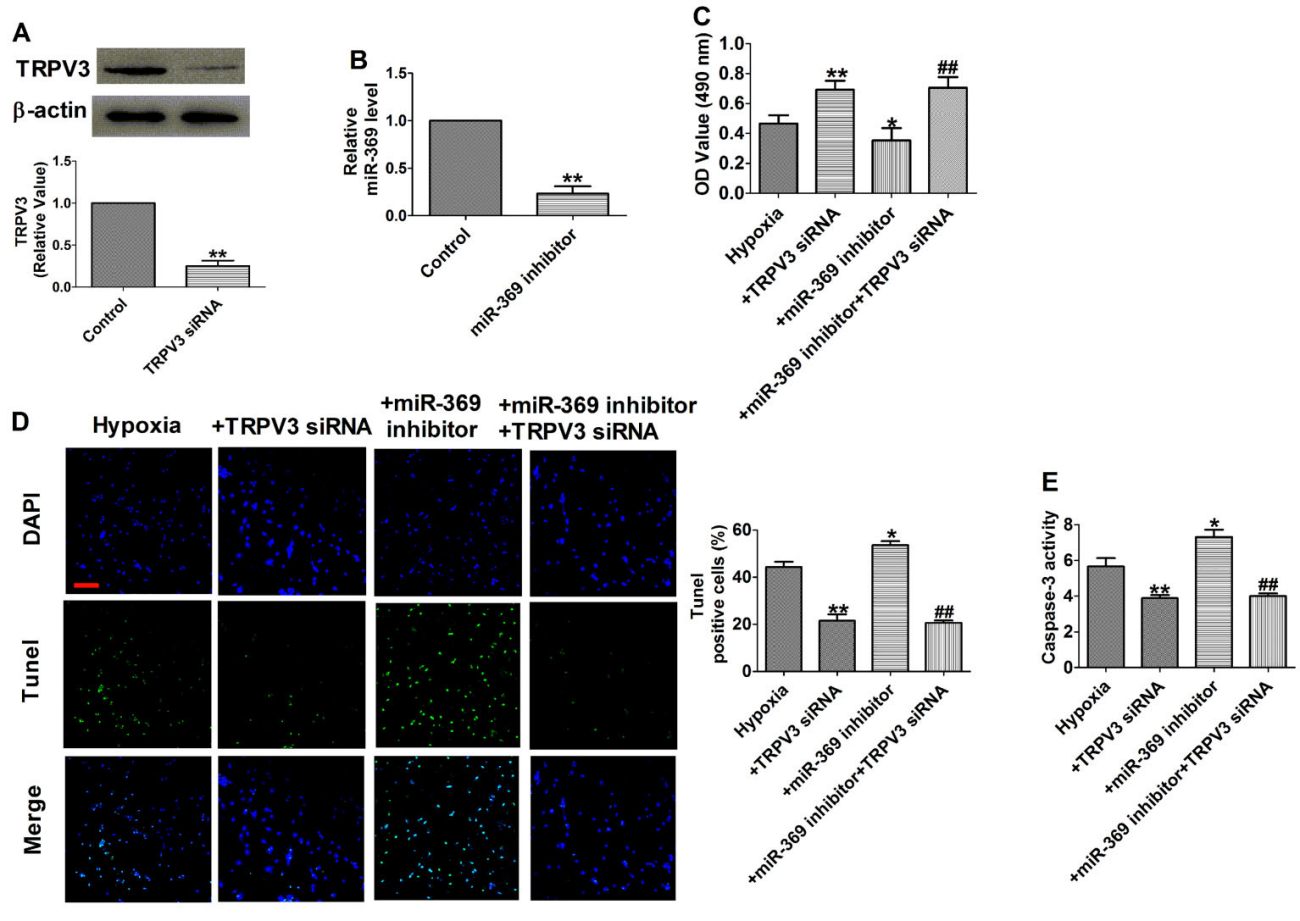
**A**, Expressions of TRPV3 protein (n=3). **B**, Relative mRNA level of miR-369 (n=6). **C,** MTT assay (n=6). **D**, Representative images of TUNEL staining of cardiomyocytes showing the apoptotic cells (100×; scale bar, 100 μm) and statistical results of TUNEL-positive cells per field (n=4). **E**, Caspase-3 activity (n=6). Data are reported as means±SE. **A** and **B**: **P<0.01 *vs* control group; **C**-**E**: *P<0.05, **P<0.01 *vs* hypoxia group; ^##^P<0.01 *vs* hypoxia+miR-369 inhibitor and hypoxia groups (*t*-test).

TRPV3 siRNA treatment efficiently repressed the TRPV3 expression at the protein level (Figure 5A), and transfection with miR-369 inhibitor significantly reduced miR-369 expression in cardiomyocytes (Figure 5B). Moreover, TRPV3 siRNA remarkably increased cell viability and suppressed cell apoptosis induced by hypoxia treatment (Figure 5C and D), which also effectively down-regulated caspase-3 activity (Figure 5E), thus suggesting a protective effect of TRPV3 siRNA in hypoxia-stimulated cardiomyocytes. Meanwhile, miR-369 inhibitor promoted hypoxia-induced cell apoptosis that was reversed by co-transfection of TRPV3 siRNA (Figure 5C-E).

**Figure 6:**
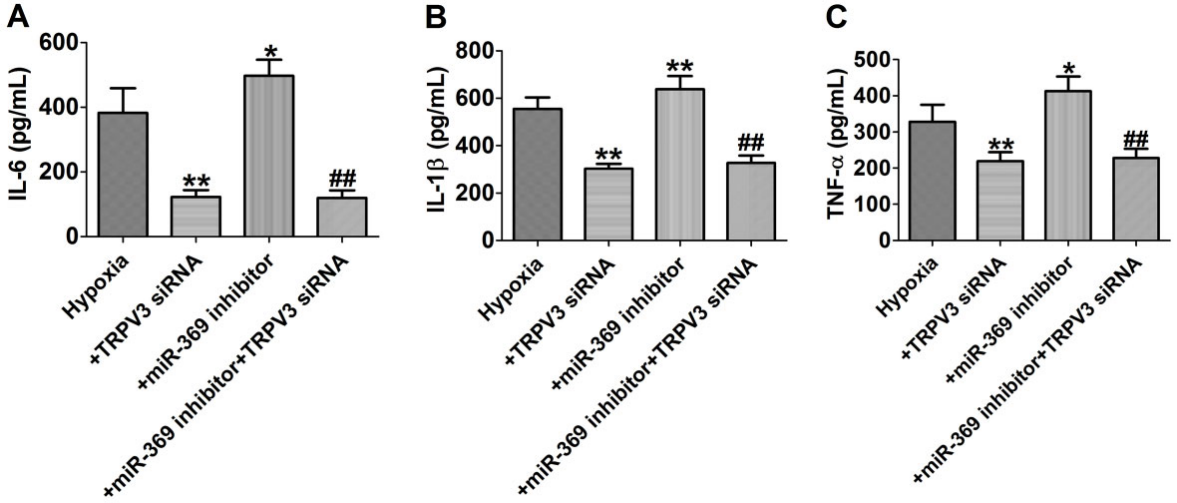
**A**-**C**, Concentrations of interleukin (IL)-6, IL-1β, and tumor necrosis factor (TNF)-α measured by ELISA assay. Data are reported as means±SE for n=4. *P<0.05, **P<0.01 *vs* hypoxia group; ^##^P<0.01 *vs* hypoxia+miR-369 inhibitor and hypoxia groups (ANOVA).

Next, TRPV3 siRNA notably decreased the secretion of the inflammatory cytokines IL-6, IL-1β, and TNF-α, indicating that TRPV3 was involved in the inflammatory response process under hypoxic conditions. Meanwhile, miR-369 inhibitor promoted hypoxia-induced inflammatory response, while co-transfection of TRPV3 siRNA reversed the inflammatory response (Figure 6 A-C). Taken together, our data demonstrated that miR-369 inhibited hypoxia-induced apoptosis and inflammatory response in cardiomyocytes by blocking TRPV3 expression.

## Discussion

The main findings of this study were: i) miR-369 was significantly reduced and TRPV3 was increased in response to ischemia/hypoxia injury; ii) miR-369 overexpression and TRPV3 siRNA attenuated cardiomyocytes apoptosis and inflammation, while miR-369 inhibition had opposite effect; and iii) miR-369 was an important regulator of hypoxia damage by targeting TRPV3.

There is growing evidence that acute MI is accompanied by an inflammatory response. The immediate inflammatory response triggered by acute MI is a key determinant of the restoration of ischemic myocardial injury to steady state (2). Increased expression of multiple endogenous inflammatory cytokines can lead to myocardial dysfunction. Cardiomyocyte apoptosis is a key cellular event in ischemic/hypoxic cardiomyopathy (22). Acute MI has been defined as myocardial cell death after severe ischemia. Inflammation is part of the response to any type of cell death. Hypoxia-induced cardiomyocyte apoptosis is an important pathological phenomenon of the heart (23). Therefore, elucidating the molecular mechanisms of cardiomyocyte inflammation and apoptosis is essential to establish a therapeutic strategy for ischemic heart disease.

Recent studies have reported that miR-369 regulates apoptosis in colorectal cancer and papillary thyroid carcinoma cells (24,25). miR-369-3p suppresses chronic inflammatory response induced by lipopolysaccharide (26). Importantly, a recent study showed that miR-369 is reduced in cardiac fibrosis tissues and miR-369 overexpression inhibits cultured cardiac fibroblasts proliferation (15). However, little is known about the role of miR-369 in hypoxia-induced cardiomyocytes apoptosis and inflammatory response. In this study, we found that overexpression of miR-369 during hypoxia treatment significantly attenuated cardiomyocytes apoptosis and inflammation, while inhibition of miR-369 caused more extensive cellular damage.

TRPV3 has been found to be a key regulator of cardiovascular diseases (17–19,27). According to our previous study, TRPV3 aggravates pathological myocardial hypertrophy through the calcineurin/NFATc3 pathway, promoting the extracellular Ca^2+^ flow and increases [Ca^2+^]i, and aggravates cardiac fibrosis through the TGF-β1 pathway (17,18). TRPV3 siRNA reduces relative fluorescence intensity of Ca^2+^ signal, leading to the decrease in the activation of cardiac autophagy and ultimately inhibiting the development of cardiac hypertrophy (19). However, little is known about the role of TRPV3 in hypoxia-induced cardiomyocytes apoptosis and inflammatory response. In this study, we have demonstrated that TRPV3 siRNA protected cardiomyocytes against hypoxia-induced apoptosis and inflammation. However, there is no direct evidence that a potential regulatory mechanism between miR-369 and TRPV3 was involved in hypoxia-induced apoptosis and inflammation of myocardial cells. In this study, miR-369 significantly decreased and TRPV3 increased in cardiomyocytes during ischemia/hypoxia treatment. Furthermore, we found that *TRPV3* was most likely a target gene for miR-369 due to the presence of the conservative sequence fragment in rat species. This is fully supported by the luciferase reporter gene and protein expression detection assays, which showed that *TRPV3* served as a direct target for miR-369. Moreover, TRPV3 siRNA reversed the role of miR-369 inhibitor in promoting cell apoptosis and inflammation in hypoxia-induced cardiomyocytes, which proved that miR-369 negatively regulated TRPV3. However, it is undeniable that, in addition to our findings, the effects of miR-369 on myocardial apoptosis and inflammation were mediated by a variety of mechanisms and the exact function of miR-369/TRPV3 in regulating hypoxic injury remains to be studied *in vivo* using animal models.

In summary, our research indicated that miR-369 is a hypoxia-reactive miRNA. miR-369 overexpression protected cardiomyocytes against hypoxia-induced apoptosis and inflammation through down-regulation of TRPV3.
